# General practitioners’ perspectives on targeted breast ultrasound as primary diagnostic test in women with focal breast complaints: An interview study

**DOI:** 10.1016/j.heliyon.2024.e40123

**Published:** 2024-11-05

**Authors:** C.C.N. Siebers, L. Appelman, M. Palm, J.C.M. Van Zelst, R.M. Mann

**Affiliations:** a Department of Medical Imaging, Radboud University Medical Center, Nijmegen, the Netherlands; b General practitioner at General Practice Geffen, Geffen, the Netherlands; c Department of Radiology, the Netherlands Cancer Institute, Amsterdam, the Netherlands

## Abstract

**Introduction:**

The high diagnostic accuracy of ultrasound opens possibilities to shift towards an initial ultrasound approach for the evaluation of focal breast complaints in women, with only additional DBT in case of unclear or suspicious ultrasound findings. As general practitioners (GPs) are important stakeholders in the diagnostic pathway, this study focuses on GPs perspective on ultrasound as primary diagnostic imaging test, as well as the GP referral process.

**Methods:**

Sixteen Dutch GPs were interviewed on the referral process and their perceived barriers and facilitators of initial ultrasound diagnostics in women presenting with focal breast complaints. Interview data were transcribed verbatim and analyzed thematically.

**Results:**

Thematic analysis identified themes related to 1) the routine breast consult (*consult characteristics*, *referral decision*, *referral rationale* and *diagnostic imaging decision*) and 2) considerations of an ultrasound-only approach. Regarding the latter, the theme *diagnostic workflow* emphasizes GPs concerns regarding long waiting times for ultrasound. *Professional communication* describes communicational barriers on patient-radiologist and radiologist-GP level. In the theme *doctor-patient relationship* shared decision-making was highlighted, while concerns existed on the lack of patient return to the GP after result disclosure at the hospital. *Effectiveness of imaging* is associated with GPs’ acceptance of the diagnostic performance of ultrasound as stand-alone modality. *Personal expertise and workload* is related to consequences of an initial ultrasound approach on GPs workload and professional tasks. Regarding the *patient benefit-harm trade-off*, various patient (dis)advantages were highlighted. *Decentralization of diagnostic evaluation* was considered a potential practical implication of ultrasound as primary diagnostic test.

**Conclusion:**

Participants seemed to welcome ultrasound-only diagnostics for the evaluation of women's focal breast complaints, emphasizing multiple benefits for both the patients and GPs. They, however, also addressed various challenges that should be taken into consideration when actually practicing an initial ultrasound approach.

## 1 Introduction

Women visiting a general practitioner (GP) with breast problems are common. Each year, around 3 % of all visits by female patients are related to breast symptoms or concerns [1,2]. The most common problems are breast lumps and breast pain (8.8 and 14.1 per 1000 women respectively), but nipple complaints, and fear of breast cancer are also frequently occurring (2.5 and 3.6 per 1000 women respectively). While the latter complaints are only associated with a small risk of breast cancer (1–2 %), cancer is the underlying cause in 8 % of women with breast lumps [2]. Therefore, it is crucial for GPs to adequately act on such encounters to optimize early detection of breast cancer, while patients not at risk for cancer should be quickly reassured after diagnostic imaging to prevent further anxiety.

When women present with localized breast complaints in the Netherlands, GPs are expected to follow the NHG guideline for breast cancer [3]. This standard outlines the diagnostic steps GPs should undertake, including the anamnesis, physical examination of the breasts and possibly referral for additional imaging. The current standard for imaging in patients with focal symptoms differs according to age. While mammography (or Digital Breast Tomosynthesis (DBT)) is prescribed as the initial diagnostic test of choice in women ≥30 years, targeted ultrasound (US) is used in women under 30, or when pregnant or lactating. Given recent advances in US, however, it is questionable whether DBT still represents the most appropriate breast imaging modality in symptomatic women. This was investigated in multiple studies, including the Breast UltraSound Trial (BUST) [4]. In concordance with other study results [5,6], the BUST showed that targeted US is an accurate stand-alone modality in the diagnostic setting, with a very high sensitivity (98.5 %) and specificity (90.8 %). Also, the added value of DBT for the evaluation of patients’ focal complaint is low (0.2 %), with a detection rate of 0.4 % for additional asymptomatic cancers [4]. It is, therefore, suggested to perform DBT only in case of unclear or suspect outcomes during US examination.

However, organizational changes to the health care system pose various challenges, including the imperative of delivering high-quality service, ensuring the availability of skilled professionals, and addressing financial, scientific and educational implications. Furthermore, patients’ perspectives play a crucial role in modern health, prompting exploration of their views on an US-only approach in prior research [7,8]. The perspective of healthcare professionals and their willingness to adapt are influenced by all these aspects. GPs are often the first point of contact for women with breast-related concern and are responsible for deciding on further imaging procedures. Consequently, they represent an important stakeholder in the deliberation for the future implementation of an US-only approach.

Although guidelines only recommend referral based upon certain suspect symptoms, an interview study among British GPs has shown that the reasons for referring women fell into three broad categories: 1) nature of symptoms is indicative for breast cancer, so referral is considered necessary, 2) nature of symptoms is unclear, sometimes in combination with patient anxiety, family history or medical-legal concerns, so referral is considered necessary or 3) nature of symptoms is seemingly benign, so the referral decision is mainly associated with patient anxiety or medical-legal concerns [9]. Moreover, Sollie et al. showed that 54 % of patients with a family history of breast cancer but without physical symptoms were referred for further imaging by the GP, as well as 52 % of asymptomatic women reporting fear of breast cancer [1]. These findings suggest that GPs’ referral behavior is unlikely to follow from adhering to clinical guidelines alone, but is also related to patient concerns.

Although the literature provides some insight in referral behavior of GPs in women with breast problems, the exact course of action during such consultations and the communication patterns towards patients on the nature of their complaints and the value of impending diagnostic procedures are not yet explored. In addition, as US-only diagnostics implies omission of DBT in women with benign breast symptoms, it is important to assess GPs attitude towards this development. In this study, therefore, interviews are conducted with GPs on the referral process of women with breast complaints and their perspective on US as the primary imaging modality.

## 2 Materials and methods

### 2.1 Design

Individual interviews with Dutch GPs were held using a semi-structured interview guide. The BUST has been granted an exemption from requiring formal ethical approval by the ethical committee 10.13039/100017556CMO Arnhem-Nijmegen (2016–3034). No further ethical approval for this voluntary qualitative study was required. The study was conducted according to the Declaration of Helsinki, and all subjects provided written informed consent prior to enrollment in the study.

### 2.2 Subjects

The subjects in this study were GPs working in various different general practices in the Netherlands. In order to have a diverse group of participants, GPs of both genders, of varying age, with various years of professional experience, and from different regions were approached. All participants were invited between December 2021 and March 2022. GPs were recruited through personal approach by email or phone, a call on social media, or via the personal or professional network of other participating GPs. Each participant was provided with additional information on the interview beforehand and filled out a short questionnaire and informed consent.

### 2.3 Procedure

All interviews were held in person or via the online platform Zoom Meetings and were carried out by the main researcher. The interviews lasted 30–60 min. Before each interview, participants were provided with general information on the BUST study setup. After initial exploration of GPs attitude towards this approach, concise results of the study were shown and participants were asked about their thoughts on these results. All interviews were recorded and conducted by the main researcher.

### 2.4 Data analysis

IBM SPSS Statistics 25 was used for description of patient characteristics. The interview data were transcribed verbatim and thematically analyzed in “Atlas.ti” by two researchers independently (CS and MP). An inductive approach was used by adhering to six consecutive steps: data familiarization, coding, theme development, theme review, theme definition and naming, and final analysis [10]. In case of discrepancies consensus was reached through mutual discussion.

## 3 Findings

### 3.1 Participants

A total of 16 GPs were interviewed. The participants had a mean age of 43.1 years old (SD = 10.13) and an average of 11.31 years (SD = 10.52) of experience as GP. They worked in different regions across the Netherlands, either as practice owner or acting GP, and both male and female GPs participated. Table 1 provides an overview of participants’ characteristics.

**Table 1:** Participants' characteristics.

	N	*(%)*		N	*(%)*
**Sex**			**Age**		
Male	5	*(31.3)*	30–39	9	*(56.3)*
Female	11	*(68.8)*	40–49	3	*(18.8)*
**Region**			50–59	3	*(18.8)*
Gelderland	8	*(50)*	60–69	1	*(6.3)*
Overijssel	2	*(12.5)*	**Years of experience as GP**		
Noord-Brabant	1	*(6.3)*	<5	6	*(37.5)*
Zuid-Holland	1	*(6.3)*	5–15	4	*(25.0)*
Noord-Holland	3	*(18.8)*	15–25	3	*(18.8)*
Various	1	*(6.3)*	>25	3	*(18.8)*

### 3.2 Thematic analysis

**Fig. 1:**
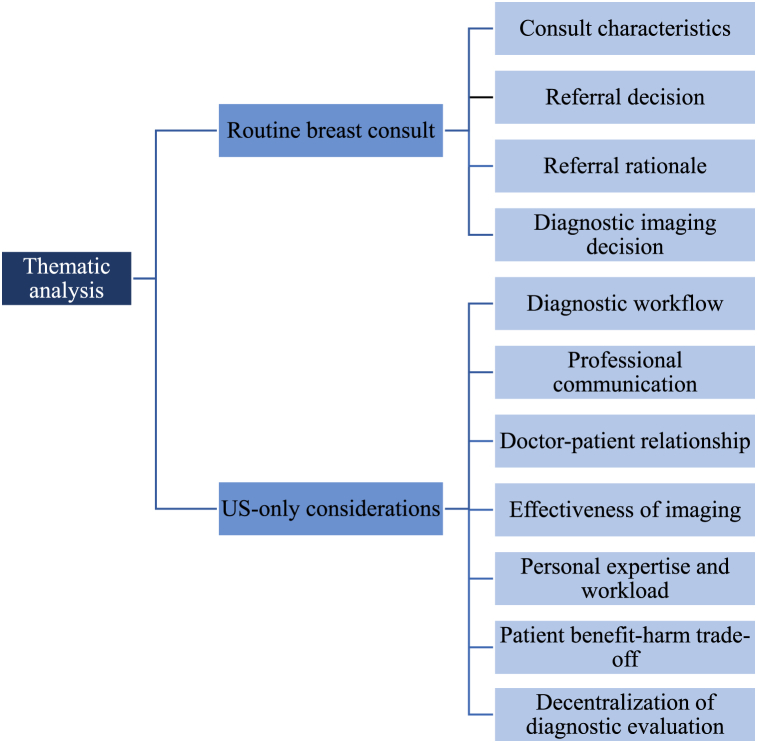
Themes as emerged from the thematic analysis.

In the interviews, two major topics were discussed: information about the routine breast consult and GPs perspective on US as primary imaging modality. Themes related to the routine breast consult are *consult characteristics, referral decision, referral rationale* and *diagnostic imaging decision*. The main themes regarding GP perspectives on US as primary test include *diagnostic workflow, professional communication, doctor-patient relationship, effectiveness of imaging, personal expertise and workload, patient benefit-harm trade-off*, and *decentralization of diagnostic evaluation*. The themes of both topics are described in Fig. 1.

#### 3.2.1 Routine breast consult

##### 3.2.1.1 Consult characteristics

First the characteristics of the GP consult with women with breast symptoms were described. Patients' age varies widely, and the complaints include various breast symptoms or fear of breast cancer. The GPs indicated that their consults are often following the NHG guideline, which includes an anamnesis and a physical examination of the patients' breasts and axillae. Therefore, the consults are always held face-to-face. During the anamnesis the GP focusses on the type of complaint, its duration, the way it was discovered and changes over time, women's family and personal history of breast cancer, the menstrual cycle, participation in the screening program and women's fear of cancer. Most GPs see women with focal breast complaints once a week to once a month, although three male GPs indicated that their female colleagues might encounter such patients more frequently than they did. Depending on the practice and women's concern, the waiting time for the consult ranges from a same-day consult to a week.

##### 3.2.1.2 Referral decision

The referral decision depends on many factors. The most important factor is whether the nature of the complaint is suspect for breast cancer. Further, GPs tend to be more reluctant to refer young women, vulnerable patients, or patients without suspect complaints (e.g. mastopathy, fluctuance with menstrual cycle or clearly normal breast tissue), while referral is considered more necessary in women with persistent complaints, a clearly palpable lump, a positive family history, or in case of GPs doubt on the nature of the complaint. Although most GPs indicated to be fairly accurate in palpating a malignant breast tumor, they also described various situations in which they were not entirely sure about their physical assessment or even misinterpreted a malignancy for a benign lesion. Another important contributor to GPs decision to refer is patients' anxiety, as fear of breast cancer tends to be ever present when consulting women with breast symptoms. Therefore, the urgency to arrange an appointment for radiological assessment is based on the degree of suspicion for breast cancer and the patients’ level of anxiety.

##### 3.2.1.3 Referral rationale

All GPs agreed that the most important reason for referring women is to get an explanation for their symptoms and to rule out breast cancer at the focal spot of the complaint. A whole-breast evaluation or evaluation of the contralateral breast with DBT is, therefore, mostly not the GPs main interest, although four participants stated that their secondary interest would be to check whether neither breasts show abnormalities and/or to provide reassurance to the patient. This is also true for patients according to the GPs; women tend to be mainly worried about their specific complaint being malignant, but incidental scenarios were described in which women did value a bilateral evaluation of the breasts.

##### 3.2.1.4 Diagnostic imaging decision

In the Netherlands, GPs refer women through a digital environment called *Zorgdomein*. They can primarily choose between the radiology department where DBT and/or US is performed and the breast clinic which is usually a section of the surgery department. However, this contrast is not that clear in every hospital, as one participant explained that *Zorgdomein* does not show separate options for each department, but they are combined under the option ‘breast diagnostics’. Three others mentioned that the option to book a breast US was absent or “hidden” in *Zorgdomein*, suggesting that sometimes radiologists or surgeons decide on the exact clinical pathway instead of the GP. Broadly, however, GPs tend to refer to the radiology department in women with symptoms of seemingly benign nature and in younger women, while referral to the breast clinic is mainly indicated in case of suspect complaints, a positive family or personal history of breast cancer, or extreme patient anxiety and quick clarity is required. In case of referral to the breast clinic, GPs do take into account the higher costs, potentially unnecessary diagnostic examinations and the burden and anxiety for patients associated with the breast clinic (n = 5). When sending women to the radiology department, the decision for DBT or US is mostly based on the NHG guideline or is left to the radiologist. However, participants mostly choose US in women <30 years, women with suspected mastitis, lactating women or when patients are very reluctant to DBT, while DBT is the modality of choice in women >30, with a positive family history, and when they are extremely worried and have a strong preference for a bilateral breast check. As the waiting time for DBT tends to be shorter than for US, the choice for DBT may also be logistically motivated. In other words, GPs do sometimes deviate from the guidelines and make a request for US instead of DBT, additional US or unilateral DBT.

#### 3.2.2 GPs considerations of an US-only approach

##### 3.2.2.1 Diagnostic workflow

The first GP consideration of an US-only approach is associated with the diagnostic workflow. There was a reported duration until a DBT exam at the hospital of two days to a week (sometimes even less when the GP could plan the appointment), while this is generally one to two weeks for US. Some GPs (n = 5) were concerned that initial US would thus increase waiting times. One GP suggested, therefore, to reserve dedicated breast US time slots.

“The thing I am most worried about is that ultrasound must be performed by a radiologist and the ultrasound waiting time is currently three to four weeks already. If these kind of ultrasounds get on top of that, I just don’t know whether that will work logistically. And I think that the thing I strive for most is that people know something as quickly as possible.”

At the breast clinic, all diagnostic examinations are performed at the same day. At the radiology department, however, GPs report that it varies whether women undergo DBT and US (including biopsy when necessary) in a same-day diagnosis trajectory, or are recalled to the hospital for further evaluation after initial DBT. Also, there appeared to be some misconception on the disclosure of the diagnostic results to the patients; some GPs noticed that, depending on the radiologist, results are sometimes already delivered at the hospital (n = 6), while other participants believed that patients should always inquire about the imaging outcomes via their GP (or in their online file) (n = 8).

“Sometimes they [patients] do not call anymore, so I think they already heard it then [at the hospital].”

“That depends on the radiologist. One is very talkative and the other does not say anything. They say, just call your GP.”

This implies that women sometimes have to wait several days for their final result. Therefore, 11 GPs expressed their preference for an initial US-approach because of the direct clarity radiologists can provide during US, as well as their greater expertise and ability to explain the conclusions best. This entails, however, some caveats. Also, four participants do prefer a return of patients to the GP because of relational purposes. This is described in *Doctor-patient relationship.*

“I just think it’s very pleasant that if you send people home from the hospital, they can go home without worries and do not sleep badly another night awaiting an outcome.”

##### 3.2.2.2 Professional communication

This theme highlights all communicational aspects. Participants described how they are transparent to patients about the apparent nature of their complaint; when suspicious, GPs express their worry and instruct women on referral to the breast clinic and when likely benign, they reassure women of the expected absence of breast cancer and explain that diagnostic imaging is done to obtain a definitive answer. Other GPs inform women in advance that, irrespectively of the suspicion of the complaint, they will get referred for imaging:

“In this way I’m covering myself, so that when I’m going to palpate and I say, oh we should take an X-ray, women will not immediately think, oh it is cancer! See!”

The amount of information GPs currently provide on which examinations are to be performed and the exact procedures tends to vary with their own knowledge and what they consider necessary to share.

“I do not explain a lot about how a mammogram goes, because actually I do not know exactly. I’ve never seen it being performed on a patient.”

Participants recognize that a targeted US might require a different way of informing patients; some would explicit that with performing only an US there is a minimal chance a non-symptomatic cancer elsewhere remains undetected, while others would not elaborate on the clinical performance at all. As one GP suggested, automatic printing of patient information when making a referral would be helpful to inform patients on their hospital visit.

“You know, [I would say] we’ll do an ultrasound and if nothing comes out, you are 99,8% sure that nothing’s there. If you want 100% certainty, we’ll have to do mammography as well.”

“I don’t think I would give any explanation in advance. That would also nullify the whole idea, you know. Look, there’s not one examination that captures everything you want to catch …”

In the current pathway, patients are often instructed to call their GP for the radiological report. However, should this be supplemented by disclosure of imaging findings during US, four GPs emphasize the importance of comprehensible language use by the radiologist, as this is currently often lacking.

“But it should just be in layman’s terms, because if you get all these explanations about calcifications people come back totally confused. Or about lumps that are currently not harmful but still have to be checked once again. Yeah, I think a lot of doctors at the hospital do not necessarily use simple language …”

It was noted that another barrier to conveying findings to patients at the hospital would be the delayed feedback to the GP. Quickly informing patients’ GP of the radiological outcomes is considered essential (n = 4).

“Something is seen which isn’t good and then an hour later the patient calls us while we don’t even know anything yet.”

It was also emphasized that, when women hear about the diagnostic conclusions via the radiologist, clear arrangements should be made on each professional's responsibilities to avoid miscommunications (n = 3).

“So at the moment there will be information, it is actually best that the responsibility also goes to the radiologist. He has already said something about it, than he can also finish it. Or he should not say anything and it comes back to me and I can proceed. But if there are two captains at one ship […] it is likely there will be miscommunication.”

GPs consider aftercare for patients important; they refer to informative websites, provide instructions on future self-examination and patient return in case of persistent complaints, and give advice on anticonception or pain medication (n = 13). One GP skepticized whether such advice would also be given at the hospital:

“In fact, that patient enters the radiology [department] with a fairly limited question […] and if only that outcome would be communicated […] I wonder whether that moment of disclosure would be so appropriate.”

##### 3.2.2.3 Doctor-patient relationship

Theme three describes GPs thoughts on an US-only approach on relational level. It stood out that the GPs tend to be very concerned with their patients and their wellbeing. Also, some participants emphasized the importance of shared decision making between patient and GP (n = 4):

“I want to avoid being a rigid doctor that says, listen, this is not how we’re going to do it, we’ll do it this way, and then half a year later you have cancer. Then your whole doctor-patient relationship is gone.”

Participants indicate it is patients' own responsibility to call their general practice for the imaging outcomes (n = 7), which is usually handled by the doctor's assistant (although GPs contact women themselves in case of a malignant outcome). This means there is mostly no “real-life” retour of patients. However, GPs that do wish to have contact with their patients after the breast evaluation would be concerned that patients are not sent back to their GP when final radiological findings are delivered by the radiologist. They find this problematic, as the radiology professionals are not familiar with the broader context of patients' complaints. Therefore, some participants (n = 4) prefer no disclosure of US findings at the hospital but via the GP instead, or at least a patient return to the GP after the hospital visit.

“I would like to put in a sort of disclaimer somewhere, like […] maybe you should discuss it further with your GP, because I do not know your story. […] Because in the end, we are the GP and thus the head practitioner. […] So you can say it [the imaging result], that is also pleasant for the patient, but it should not be a final conversation. That belongs to us.”

##### 3.2.2.4 Effectiveness of imaging

This theme is associated with the clinical performance consequences of an US-only approach. First, three GPs wondered whether a bilateral “whole-breast” US would be performed in case of an US-only approach. Moreover, GPs raised questions on the lesion detectability of both modalities:

“… it’s a bit of a gradual line how reliable it [US] is, probably per age, depending on the tissue … I don’t know, I would feel like it is only reliable in younger women and less later on.”

“I always learned that the mammogram is no holy grail, that there can also be misses now and then. We also have a number of such nasty cases in our practice.”

Participants frequently asked about the sensitivity of DBT and US combined, and they underscored that an US-only approach should not detract from the current clinical performance (n = 6). Also, it was emphasized that their most important interest was reassurance of women about the absence of breast cancer.

“That patient has to come back to me and I should be able to say, more or less, you don’t have breast cancer.”

During the interviews, participants were shown results of the clinical impact of the BUST approach. Most of them believed the number of asymptomatic incidental findings detected with DBT was acceptable (n = 12), especially as these tumors were regarded as by-catch and half would be picked out in the national screening program (although it was noted that participation is never guaranteed). It was, however suggested by one GP to further specify the missed lesions to determine certain risk groups that could benefit from additional DBT. Also, concerns were raised on the false-negative US findings, and the importance of radiologists’ US skills to avoid such findings was emphasized (n = 5). Still, the majority of the participants indicated to be in favor of an US-only approach based on the result of the BUST (n = 12).

“Indeed, the question is: would you want to spare women the nasty exam of mammography so that they do not have to undergo it, but than there is the chance that something comes out anyway …”

“ […] I think the most important thing about ultrasound is the variability within assessors and that you will need a good radiologist to assess it.”

##### 3.2.2.5 Personal expertise and workload

This theme highlights all personal consequences for GPs of performing initial targeted US. It stood out that participants sometimes argued about an US-only approach from their own frame of reference, e.g. because they underwent US or DBT themselves, because of their limited experience as GP or because of breast cancer experiences in their personal environment (n = 4). Also, eight participants expressed their lack of knowledge on the diagnostic procedures in the hospital, as it is hard to stay informed of all the guidelines. One GP, therefore, suggested to enter the US-only protocol in *Zorgdomein*.

“[It] would feel more pleasant for me to send them to an exam that I know and can explain to them, and that I know is not very burdensome … that women do at least not undergo an unpleasant exam because of my inexperience.”

It was also put forward that clear instructions should be included in the NHG guideline on the targeted nature of the US exam and in which situations to refer for US only, as it was noted that discussion could arise on patients' complaints’ being either targeted or more diffuse (n = 4). One GP raised concerns about medico-legal matters as a result of unclear regulations:

“There should be as little discussion as possible. Discussion in the sense of that we’re getting disciplinary cases about ‘you should’ve requested a mammogram’ …”

Severel personal advantages of the US-only approach were described. It is a targeted exam and thus feels more purposeful for GPs for assessment of the complaint (n = 4). Besides, it would lower the treshold for GPs to refer women for additional imaging as US is less stressful than DBT (n = 4). Four participants also noted that explanation of findings by the radiologist could result in less patient consultations for the GP. Five others, however, stressed that patients might not be sufficiently satisfied with only a targeted evaluation of their complaint, therefore returning to their GP.

“And for me maybe, if it would happen at a larger scale, that I would not have to do it [disclose results]. Because we’re already very busy …”

##### 3.2.2.6 Patient benefit-harm trade-off

Patient benefit-harm trade-off is related to concerns of the patients when US would replace DBT as initial imaging test. GPs described that women's knowledge on the diagnostic breast tests is generally low and patients tend to rely on the decisions of the medical professionals (n = 7).

“People have no idea. Ultrasound, mammogram … they have no idea. They want to go to the hospital, because that’s where they figure it out.”

Two patient disadvantages of DBT were frequently mentioned by GPs; the X-radiation (n = 5) and the burden of the exam (n = 16). Five GPs describe the high patient reluctance towards DBT that raises the treshold to undergo diagnostic imaging, with some patients even asking for an US in advance (n = 2). Participants, therefore, expect that most patients would prefer diagnostics with US only, because of the accessiblity and low burden of US, the absence of X-radiation, lower costs, and the personal and transparant interaction with the radiologist. Also, quick reassurance can be provided during US.

“That someone can watch along and that a sonographer can explain ‘well here you can see this, this and that’, of course that does’t happen at mammography.”

However, GPs noted that patients can be extremely anxious and a bilateral breast evaluation with DBT can help reassure them (n = 5). When only performing US, this could potentially leave patients with a feeling of having undergone sub-optimal breast asssessment. Four participans indicated that they might thus request additional DBT in case of extreme patient worry. Also, when thinking about the malignant additional findings detected with DBT, GPs were prone to put themselves in the patient's shoes (n = 4).

“Well I do think they will get insecure whether enough is seen on an ultrasound, because they always got that mammography. So why not anymore?”

“If I look at this [additional malignant DBT findings], I would think ‘what if you were that one person’ …”

##### 3.2.2.7 Decentralization of diagnostic evaluation

A potential practical implication of an US-only approach could be decentralization of diagnostic breast assessment. This would imply performance of a breast US at the general practice or another nearby location by a skilled sonographer. GPs describe this to be advantageous because no referral to a hospital is needed (n = 3), it is convenient and more accessible for patients (especially in rural areas) (n = 8), women can undergo it quickly and be immediately reassured in case of normal findings (n = 8), and it could potentially save costs (n = 2).

“What I also see sometimes at for example taking blood […] something like that is even more accessible, because that’s nearby.”

A downside would be that no follow-up diagnostics are possible in case of suspect US findings (n = 3), while on the other hand GPs run the risk to let women undergo US too easily (n = 1). Moreover, one GP noted that decentralization could lead to even longer waiting times:

“In practice, too few people could be gathered and then of course you get a bit of waiting time, because than you have to pool people and some have to wait longer.”

GPs also described their own ability to assess US. Although virtually no participants could perform US themselves, in some general practices it was discussed to purchase an US machine and offer courses for GPs (n = 7). Regarding breast US, however, GPs mainly emphasized the complexity of interpreting breast lesions and the associated risks in case of misinterpretation (n = 12). Also, this would imply a lot of upskilling and time investment (n = 9), while the frequency of patients with breast symptoms is generally too low to gain experience (n = 11). Moreover, four participants mentioned the high costs of an US device and the difficulty in defining the types of US to be performed by GPs (n = 3).

“I’m a bit hesitant, because I think … yeah what kind of ultrasound do you do, because that’s actually a profession in itself, sonographer … You do not learn that in a two-evening course, and what kind of … Do you only use it to check whether the IUD is in place or are you also looking at gallstones or are you indeed also going to assess the breasts?”

## 4 Discussion

In this interview study, we explored GPs views on the referral process of women with breast complaints and their perspective on US-based diagnostics. Participants seemed to welcome an US-only approach, emphasizing multiple benefits for patients and GPs. However, they also addressed various different challenges on logistical, communicational, relational, diagnostic, personal, patient and practical level.

GPs referral decisions do not seem to follow from adhering to protocols alone. Ruston also described that clinical guidelines are solely based on scientific knowledge, while GPs largely utilize their ‘tacit knowledge’ to support referral decisions, based on their beliefs and personal and anecdotal experiences [9]. Moreover, congruent with our study results, this is affected by patient wishes, anxiety, and pressure [[11], [12], [13]].

The interviews revealed that patient communication and education about their breast cancer risk and the diagnostic exams, as well as disclosing imaging outcomes, are major tasks of GPs. Involving radiologists in providing US findings to patients, however, fits into the broader goal of moving from the traditional role of radiologists transmitting imaging findings to the GP without having direct patient communication, to patient-centered radiology which involves more patient-radiologist communication [14]. Should the results be provided by the radiologist, the importance of direct feedback to the GP was highlighted. This issue has been raised before; when patients immediately consult their GPs while they are not yet informed about the outcome, this could negatively affect the GP-patient relationship, as well as the mutual physician relationship. Therefore, clear arrangements should be made on who conveys which information to patients [15].

GPs expressed their concern for increased waiting times when performing initial US. This is important, as patients think a short length of time to undergo diagnostic imaging is essential [16]. Therefore, GPs consider quick reassurance for patients by delivering results during US a great asset. It was, however, pointed out that not all radiologists are inclined to convey imaging conclusions. This is possibly due to time constraints [17]. It has been shown that the average time spent on delivering US results to patients is 2 min for normal findings, 5 min for minor abnormalities and 8 min for significant abnormalities [18]. However, as the vast majority of women with breast complaints presents with normal or benign US findings, the time spent on communicating results would remain limited [4].

Another explanation for radiologists’ reluctance to communicate image results to patients could be related to the fear to interfere with the tasks of the referring physician [19]. Indeed, it is shown that 76–85 % of referring physicians believe radiologists should disclose normal findings, while this is true for only 44–58 % in case of abnormal findings. This is likely related to GPs perception that radiologists are not sufficiently equipped to deal with the emotional impact of malignant diagnoses, in contrast to the GP who holds an established relationship with the patient [20,21]. Still, previous study results show that 94 % of patients wish to hear their US findings from the performing specialist [18], while for women undergoing mammography this is true for 93 % and 90 % in case of normal and abnormal findings respectively [22].

The majority of our participants would be open to US-based diagnostics in case of solid scientific evidence. Accordingly, results from another GP interview study suggest that as long as the scientific basis of the guideline is clear and accepted by the target group, this would encourage adherence to the guideline [23]. However, some participants mentioned they might still order a DBT exam in case of extreme patient anxiety or preference for additional DBT. This is in line with findings from earlier study results that emphasize how patient demands highly affect the performance of unwarranted imaging [9,23]. Another known barrier to GP adherence to guidelines is lack of clarity on the guidelines [23]. It was mentioned that GPs run the danger of ordering an inappropriate breast exam due to unclear regulations. This fear could result in GPs practicing *defensive medicine*, which is defined as “ordering of treatments, tests, and procedures for the purpose of protecting the physician from criticism rather than diagnosing or treating the patient” [24]. To avoid GPs ordering additional DBT out of such a defensive stance, clear instructions on which diagnostic tests to perform in which situation are warranted.

It is known that GPs experience very high workloads, especially in the Netherlands due to a greater diversity of duties compared to other European countries [25]. Should an US-only approach limit the amount of patient-GP consultations, this could positively impact the GP workload. Another advantage of performing US only for GPs is that unnecessary discomfort is avoided, which is also related to GPs perceived patient benefits: it was expected that most patients would prefer diagnostics with US only because of the absence of X-radiation and pain, the low costs, the personal patient-radiologist interaction and quick reassurance on the absence of breast cancer.

Decentralization of imaging is a potential practical implication of an US-only approach. As there are increased possibilities to transport radiologic equipment to patients and provide imaging services closer to home, basic examinations such as breast US could be established in larger general practices or other primary care centers. This can especially be valuable in rural areas to minimize the distance between patients and hospital. However, performing decentralized imaging examinations requires the availability of state-of-art equipment and trained staff members who should be able to perform a sufficient number of examinations to maintain their radiological skills and achieve cost-effectiveness of this approach [26]. However, the alteration of clinical pathways presents numerous other challenges to the health care system besides the requirement of skilled professionals, including the usual quality-demands, and financial, scientific and educational concerns. Given the dynamic nature of the evolving work landscape and the continual refinement of optimal imaging choices, it is crucial to consider how the system can adapt accordingly. For example, the interviews revealed that radiologists, rather than GPs, sometimes decide which exams to perform. This may prompt inquiries concerning the role of GPs and which tasks could potentially be delegated to radiologists, which is particularly pertinent in the light of the increasing workload burdening GPs and the imperative to stay informed about all protocols [25]. These multifaceted challenges warrant careful consideration in the potential adoption of an US-only approach in the future.

There are several strengths to this study. It was the first study to offer a comprehensive understanding of the referral process of women with breast symptoms, alongside elucidating the benefits and challenges inherent in adopting an US-only approach for breast cancer diagnostics from the perspective of GPs. Our methodology employed a representative sample from the Dutch GP population, encompassing practitioners of both genders, spanning diverse age groups, varying years of professional experience, and hailing from different regions throughout the country. The exploration of diverse GP perspectives has relevant practical implications, as the evaluation of both advantages and complexities associated with US-based diagnostics could inform the future refinement of breast imaging protocols.

Although this research provides useful insights into GPs perspective on the breast imaging pathway, there are some limitations to our study. First, it cannot be used to evaluate whether GPs in general would favor an US-only clinical pathway, as this would require a quantitative research design. Moreover, a limited number of GPs were interviewed, and only Dutch GPs participated in the study. As other countries might adhere to somewhat different guidelines, this study thus provides limited generalizability to the broader clinical practice of breast cancer diagnostics outside the Netherlands.

## 5 Conclusion

This study provides insight in the referral process of women with focal breast complaints and GPs views on an US-only approach for the evaluation of these women's complaints. Participants seemed to welcomeUS-only diagnostics for the evaluation of focal breast complaints due to the low clinical impact of omitting DBT from the diagnostic pathway, potential decentralization of diagnostic breast evaluation, reduced workload for GPs and various patient advantages. Concerns, however, existed on long US waiting times and personal factors such as medico-legal issues, the inability to stay informed of all guideline updates and heightened time investment in case of patient return. Further highlighted barriers included communicational and relational issues on patient-radiologist and radiologist-GP level, such as delayed feedback of imaging reports and confusion about professional tasks, lack of simple language and aftercare by radiologists, and no patient return to the GP to close the circle of care. Should the guidelines shift towards an initial-US approach for the evaluation of women with breast complaints, it is important to take these facilitators and barriers into account.

## CRediT authorship contribution statement

**C.C.N. Siebers:** Writing – original draft, Visualization, Supervision, Project administration, Methodology, Investigation, Formal analysis, Data curation, Conceptualization. **L. Appelman:** Writing – review & editing, Methodology, Funding acquisition, Conceptualization. **M. Palm:** Writing – review & editing, Formal analysis. **J.C.M. Van Zelst:** Writing – review & editing, Methodology, Conceptualization. **R.M. Mann:** Writing – review & editing, Supervision, Methodology, Funding acquisition, Conceptualization.

## Ethics declarations

The BUST has been granted an exemption from requiring formal ethical approval by the ethical committee 10.13039/100017556CMO Arnhem-Nijmegen (2016–3034). No further ethical approval for this voluntary qualitative study was required. The study was conducted according to the Declaration of Helsinki, and all subjects provided written informed consent prior to enrollment in the study.

## Data availability statement

The raw data for this study are interviews transcripts, which may contain sensitive participant information. GPs did not give explicit consent for sharing this information in the public domain. Hence, we are unable to publicly share this data. Relevant de-identified excerpts of the transcripts are included in the paper. Additional data requests for researchers who meet the criteria for access to confidential data can be sent to the METC Oost-Nederland (WMO) (contact via METCoost-en-CMO@radboudumc.nl).

## Funding declaration

The BUST was supported by a 10.13039/501100001826ZonMw grant (number 843002823) rewarded to R.M. Mann. The funder provided support in the form of salaries for authors C.C.N. Siebers, L. Appelman. and M. Palm, but had no role in study design, data collection and analysis, decision to publish, or preparation of the manuscript.

## Declaration of competing interest

The authors declare the following financial interests/personal relationships which may be considered as potential competing interests: C.C.N. Siebers reports financial support was provided by 10.13039/501100001826ZonMw. L. Appelman reports financial support was provided by 10.13039/501100001826ZonMw. M. Palm reports financial support was provided by 10.13039/501100001826ZonMw. If there are other authors, they declare that they have no known competing financial interests or personal relationships that could have appeared to influence the work reported in this paper.
