# Supplementary material for: General practitioners’ perspectives on targeted breast ultrasound as primary diagnostic test in women with focal breast complaints: An interview study
Source: Heliyon. 2024 Nov 5;10(22):e40123. doi: 10.1016/j.heliyon.2024.e40123 (PMC11583702; doi:10.1016/j.heliyon.2024.e40123)
Supplement: Multimedia component 1 [file mmc1.docx]

**Supplementary file A. Interview guide**

1. How does a consult with patients with breast complaints go?

- How often do you see such patients in your practice?
- Do you always see the patients face-to-face?
- Do you always perform a physical examination?
- What do you tell the patients?

1. On what basis do you decide to refer patients for diagnostic imaging?
2. What is your indication when you refer patients for diagnostic imaging?

- What does that depend on?
- Does that differ among certain patients (e.g. older/younger women, positive family history, preference patient, type of complaint etc.)?

1. What is patients’ rationale for visiting the GP?

- What does that depend on?
- Does that differ among certain patients (e.g. older/younger women, positive family history, preference patient, type of complaint etc.)?

1. Do you speak to or see the referred patients after imaging at the hospital?

- Who delivers the imaging results to the patients?
- How much time is there between the imaging and delivery of results to the patients?
- What is your personal preference regarding the delivery of results (at the hospital or via the GP)?
- How does an ultrasound-first approach affect this?

1. Do you/are you able to perform ultrasounds yourself as a GP (specifically breast ultrasound)?
2. How would you feel about omitting the mammogram after a benign ultrasound in patients that you referred to the hospital?

- What would be the advantages?
- What would be the disadvantages?
- What would be other potential consequences for you as a GP?

1. How do you think patients would feel about omitting the mammogram after a benign ultrasound?

*Presenting BUST results*

1. How do you now feel about omitting the mammogram after a benign ultrasound?
2. How do you now think patients would feel about omitting the mammogram after a benign ultrasound?
